# Supplementary material for: Why Did All Patients with Atrial Fibrillation and High Risk of Stroke Not Receive Oral Anticoagulants? Results of the Polish Atrial Fibrillation (POL-AF) Registry
Source: J Clin Med. 2021 Oct 8;10(19):4611. doi: 10.3390/jcm10194611 (PMC8509343; doi:10.3390/jcm10194611)
Supplement: Supplementary file 1 [file jcm-10-04611-s001.zip › jcm-1387494-supplementary.pdf]

Table S1. Factors associated with the selection of an OAC over no OAC for stroke prevention in patients with AF: univariable logistic regression models.

Factors	OAC/No OAC		
	**OR**	**95%CI**	***p***
Age	0.98	0.97–0.99	0.001
Female	0.90	0.71–1.14	0.402
**Type of atrial fibrillation**			
Paroxysmal AF	0.90	0.71–1.14	0.393
Persistent AF	1.84	1.32–2.56	<0.001
**Medical history**			
Hypertension	1.53	1.1–2.10	0.009
Heart failure	0.96	0.74–1.24	0.748
Vascular disease	0.81	0.63–1.03	0.090
Coronary artery disease	0.81	0.61–1.03	0.097
Previous myocardial infarction	0.65	0.51–0.84	0.001
Peripheral artery disease	0.68	0.51–0.91	0.009
Previous stroke/TIA/peripheral embolism	0.33	0.21–0.52	<0.001
Diabetes mellitus	0.88	0.69–1.11	0.283
Any previous bleeding	0.21	0.14–0.31	<0.001
Previous intracranial bleeding	0.15	0.07–0.32	<0.001
Cancer	0.35	0.24–0.51	<0.001
Hemoglobin <12 g/dL	0.48	0.38–0.61	<0.001
eGFR < 60 mL/min/1,73 m^2	0.69	0.54–0.87	0.002
CHA_2DS_2VASc score	0.97	0.90–1.05	0.455
HAS-BLED score	0.83	0.73–0.96	0.009
**Reason for hospitalization**			
Electrical cardioversion	7.67	4.28–13.73	<0.001
Planned coronarography/ PCI	0.92	0.63–1.33	0.653
Planned CIED implantation/reimplantation	1.89	1.14–3.13	0.013
Acute coronary syndrome	0.39	0.27–0.56	<0.001
Heart failure	0.87	0.66–1.15	0.323
Ablation other than AF	0.94	0.56–1.57	0.811
AF without any procedures	1.55	0.84–2.89	0.163

Abbreviation: AF, atrial fibrillation; CIED, cardiac implantable electronic device; eGFR, estimated glomerular filtration rate; IQR, interquartile range; OAC, oral anticoagulant; PCI, percutaneous coronary intervention; SD, standard deviation, TIA, transient ischemic attack. CHA_2DS_2-VASc score: congestive heart failure (1 point), hypertension (1 point), age ≥ 75 years (2 points), diabetes mellitus (1 point), stroke/TIA/thromboembolism (2 points), vascular disease (1 point), age 65–74 years (1 point), female sex (1 point). HAS-BLED score: hypertension (1 point), liver disease (1 point), renal disease (1 point), stroke history (1 point), bleeding history (1 point), labile INR, age > 65 years (1 point), drug (concomitant use of NSAID or antiplatelet agent, 1 point).
